# Isolation of mouse mesenchymal stem cells with normal ploidy from bone marrows by reducing oxidative stress in combination with extracellular matrix

**DOI:** 10.1186/1471-2121-12-30

**Published:** 2011-07-06

**Authors:** Guokuan Fan, Lai Wen, Minshu Li, Chao Li, Benping Luo, Fang Wang, Lingjun Zhou, Lin Liu

**Affiliations:** 1 School of Life Science, Sun Yat-Sen University, Guangzhou 510275, China; 2 College of Life Sciences, Nankai University, Tianjin 300071, China

## Abstract

**Background:**

Isolation of mouse MSCs (mMSCs) with normal ploidy from bone marrow remains challenging. mMSCs isolated under 20% O_2 _are frequently contaminated by overgrown hematopoietic cells, and could also be especially vulnerable to oxidative damage, resulting in chromosomal instability. Culture under low oxygen or extracellular matrix (ECM) improves proliferation of MSCs in several species. We tested the hypothesis that culture under low oxygen in combination with ECM prepared from mouse embryonic fibroblast (MEF-ECM) could be used to purify proliferative mMSCs, and to reduce oxidative damage and maintain their chromosomal stability.

**Results:**

Optimization of culture conditions under 20% O_2 _resulted in immortalization of mMSCs, showing extensive chromosome abnormalities, consistent with previous studies. In contrast, culture under low oxygen (2% O_2_) improved proliferation of mMSCs and reduced oxidative damage, such that mMSCs were purified simply by plating at low density under 2% O_2_. MEF-ECM reduced oxidative damage and enhanced proliferation of mMSCs. However, these isolated mMSCs still exhibited high frequency of chromosome abnormalities, suggesting that low oxygen or in combination with MEF-ECM was insufficient to fully protect mMSCs from oxidative damage. Notably, antioxidants (alpha -phenyl-t-butyl nitrone (PBN) and N-acetylcysteine (NAC)) further reduced DNA damage and chromosomal abnormalities, and increased proliferation of mMSCs. mMSCs isolated by the combination method were successfully used to generate induced pluripotent stem (iPS) cells by ectopic expression of Oct4, Sox2, Klf4 and c-Myc.

**Conclusions:**

We have developed a technique that allows to reduce the number of karyotypic abnormalities for isolation of primary mMSCs and for limited culture period by combination of low oxygen, MEF-ECM, antioxidants and low density plating strategy. The effectiveness of the new combination method is demonstrated by successful generation of iPS cells from the isolated mMSCs. However, a culture system for mMSCs still is needed to prevent all the anomalies, especially after a long-term culture period.

## Background

MSCs first described by Friedenstein [1] show great potency of differentiation into osteoblasts, chondrocytes, adipocytes, and other lineages [2], and have gained widespread use in various research fields, and created great promise for cell therapy. While bone marrow derived MSCs (also named marrow stromal cells) have been successfully isolated by plastic adherence from many species including human, bovine and rat [2-5], isolation of normal ploidy mouse MSCs (mMSCs) from bone marrow remains to be big challenging. First, mMSCs isolated by the classical method of plastic adherence are frequently contaminated by overgrown hematopoietic cells [6]. mMSCs could be purified at early passage by immunodepletion of hemaetopoetic cells, but the immunodepleted cells exhibited poor proliferation [7,8]. Other methods for purifying mMSCs require long-term culture under various conditions [9-12]. However, those isolated mMSCs were actually immortalized stromal cell lines similar to hundreds of murine marrow stromal cell lines established during the past few decades [8]. This was further proved by several studies showing that mMSCs could transform spontaneously upon *in vitro *culture [13-16].

Another characteristic of mMSCs is their high chromosomal instability [13,15-19]. Bone marrow mesenchymal stem cells *in vivo *adapt to low oxygen tension in the bone microenvironment [20]; culture under normal oxygen atmosphere (20% O_2_) may exert excess oxidative damage to mMSCs. Furthermore, mouse cells are more sensitive than human cells to oxygen [21], and immortalization of MEF under 20% O_2 _has been associated with increased oxidative DNA damage [22]. Thus, mMSCs under standard culture conditions (20% O_2_) could be especially vulnerable to oxidative damage, leading to chromosome instability and finally immortalization and transformation. Indeed, low oxygen has been consistently shown to improve proliferation of MSCs in several species including human, rat, and porcine [23-26], and also primary mMSCs [27-29].

MSCs seeded onto extracellular matrix (ECM) show enhanced proliferation. ECM or components of ECM such as laminin and collagen could increase expansion ability of human MSCs [30-32]. Hyaluronan and fibrin also improve proliferation of mouse adipose-derived MSCs and bone marrow MSCs, respectively [33,34]. Nevertheless, intact ECM created from PYS-2 cells influences proliferation of human MSCs more than its major components, laminin and type IV collagen. ECM generated from MEF (MEF-ECM) is widely used as a substrate for derivation and maintenance of human embryonic stem cell (ES) lines [35]. It would be interesting to test whether MEF-ECM affects proliferation and ploidy of mMSCs.

Initially, we attempted to isolate mMSCs under classical 20% O_2 _condition and obtained only immortalized mMSCs lines with frequent chromosome abnormalities and even with tumorigenic potentials. Following extensive experimentation, we developed an improved method to purify mMSCs with normal ploidy by seeding marrow cells directly onto MEF-ECM at 5 × 10^4 ^cells/cm^2 ^under 2% O_2_, with addition of antioxidants in the primary culture.

## Results

### Culture under 20% O_2 _leads to immortalization of mMSCs

**Figure 1:**
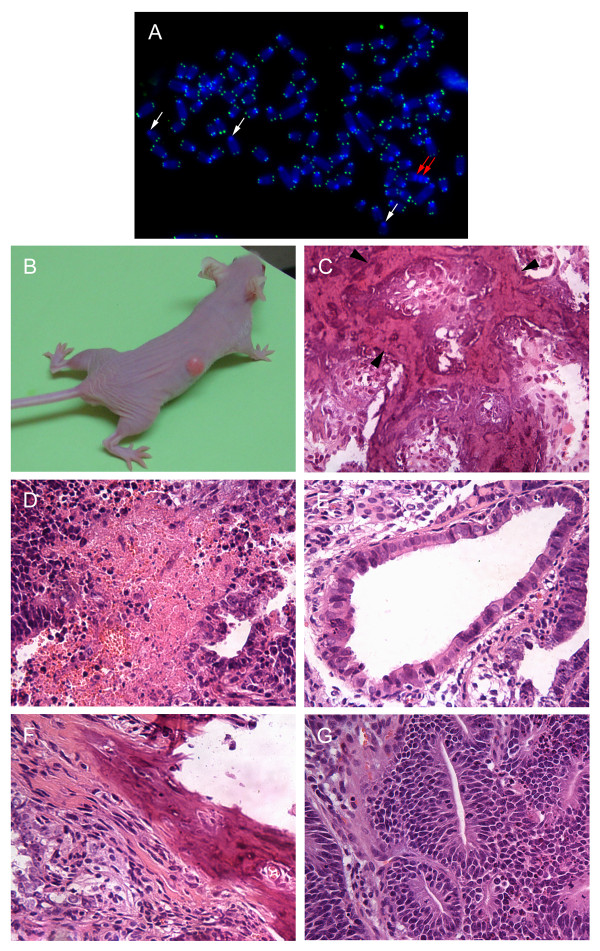
**Tumorigenesis of mMSC cell lines established under culture at 20% O_2_**. (A) Very weak telomere signals and chromosome fusions in MSC1226 at P49 revealed by FISH with the telomere-specific (CCCTAA)_3 _probe. White arrows, telomere free signals. Red arrows, end-to-end chromosome fusions. (B-C) Mineralized tumor formed by i.p. injection of 2 × 10^6 ^MSC1226 cells at P49. Black arrow heads, areas of mineralization. (D-G) Teratoma-like tumor formed by MSC0509 cells at P24. The tumor contains large areas of necrosis (D) and endoderm (E), mesoderm (F) and ectoderm (G). Tissue sections in (C) to (G) were stained with hematoxylin-eosin. Original magnification in A ×1000, in C-G ×200.

Initially, we attempted to optimize culture conditions for mMSCs under 20% O_2_. Efficiency of isolation of mMSCs was compared by culture in wells coated with gelatin or fibronectin or uncoated wells, different basal medium including DMEM and RPMI 1640, fetal bovine serum of 10-20% of various sources, a variety of growth factors including PDGF, EGF, LIF [36], or bFGF [7], and conditioned medium including MEF conditioned medium, bone marrow conditioned medium[11], and StemPro MSC SFM (Invitrogen). Under all these conditions, mMSCs ceased growth at passage 3-4 after about 3-4 weeks from the initiation of the culture. Continued culture after growth-ceasing period resulted in renewed growth and immortalization of the cells about two months in culture. Karyotype analysis of 13 such immortalized mMSC lines showed that all these cell lines exhibited extensive abnormal karyotype, mainly composed of polyploidy. Five cell lines were subjected to telomere FISH analysis with PNA probe specific for telomere repeats, and very weak telomere signals and end-to-end chromosome fusions were revealed in all cell lines analyzed (Figure 1A). Five selected cell lines with high proliferation capacity were further tested for their potential *in vivo *tumorigenicity using immunodeficient nude mice. One cell line (MSC1226, at passage 49) generated osteosarcoma-like tumor (Figure 1B, C), similar to other studies [14,15]; another cell line (MSC0509, at passage 24) showed teratoma-like tumor formation (Figure 1D-G).

### Low (2%) O_2 _improves purification of mMSCs

**Figure 2:**
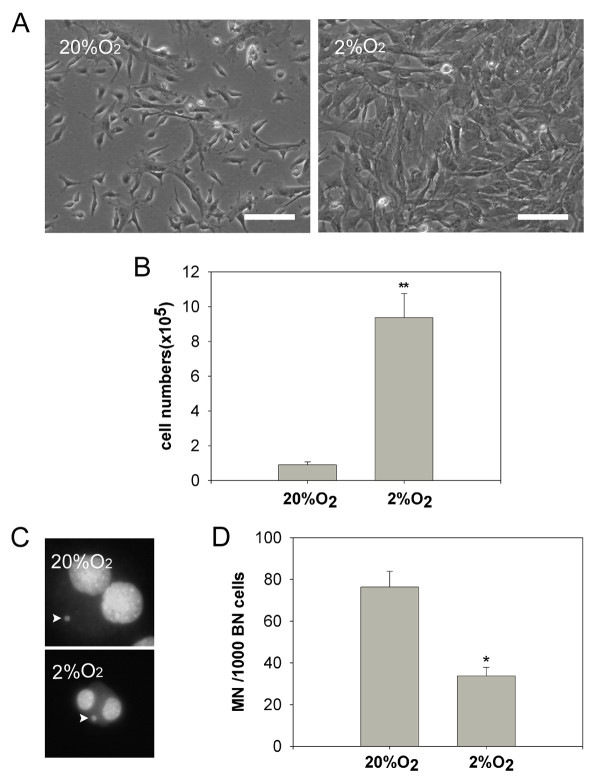
**Reduced DNA damage of mMSCs by culture under low (2%) O_2_**. (A) Morphology of primary mMSCs under 20% O_2 _and 2% O_2_, respectively. (B) Number of primary mMSCs cultured 20% O_2 _and 2% O_2_. Bone marrow cells were plated at a density of 2 x10^5 ^cells/cm^2 ^in 100 mm culture dish and cultured for 5 days under 20% O_2 _and 2% O_2_, respectively, and cells were counted by hemocytometer. (C) Micronucleus in immunodepleted mMSCs. Arrows indicate micronuclei in binucleated cells. (D) Comparison of spontaneous micronucleus formation in immunodepleted mMSCs cultured under 20% O_2 _and 2% O_2_, respectively. Data are expressed as the mean ± SD. *p < 0.05, **p < 0.01; n = 3. Scale bar = 100 μm. Original magnification in C ×400.

Consistent with previous studies [27-29], low oxygen (2% O_2_) significantly improved the proliferation of mMSCs and the cell number increased approximately ten-fold (10.5 ± 1.4, p < 0.01; n = 3.) under culture at 2% O_2_, compared to 20% O_2 _(Figure 2A, B). To understand the underlying mechanisms, mMSCs cultured under both conditions were depleted of CD45 and Ter119 positive cells and the resultant immunodepleted mMSCs (IDmMSCs) were analyzed for DNA damage by micronucleus assay [37]. Spontaneous micronucleus formation was found at higher levels in IDmMSCs cultured under 20% O_2 _than under 2% O_2 _(Figure 2C, D), indicating accelerated proliferation of mMSCs under low oxygen condition in association with reduced nuclear damage.

**Figure 3:**
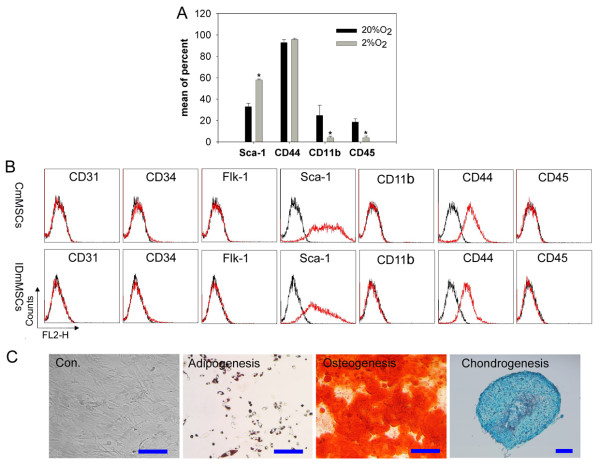
**Purification of mMSCs by culture under 2% O_2 _condition**. (A) Flow cytometry analysis of epitopes on primary mMSCs. Few cells show CD45 and CD11b antigens by culture under 2% O_2 _compared to under 20% O_2_. (B) Flow cytometry analysis of epitopes on mMSCs at passage one. CmMSCs show epitopes similar to IDmMSCs. (C) Differentiation of CmMSCs. Adipogenesis, osteogenesis, and chondrogenesis were indicated by Oil Red, Alizarin Red (pH 4.1) and Alcian blue, respectively. Scale bar = 100 μm.

More homogeneous cell population of fibroblast-like cells were found in mMSCs cultured under 2% O_2, _compared to those under 20% O_2 _(Figure 2A). Flow cytometry analysis showed that contamination by hematopoietic cells was greatly reduced by hypoxic culture. Under low oxygen, cell populations showed less expression of CD45 and CD11b antigens while maintaining high expression of Sca-1 and CD44 (Figure 3A), indicative of MSCs in most species. These results indicate that low oxygen improves purification of mMSCs, consistent with observations on rat MSCs [38].

Low density plating strategy was proved to benefit purification of mMSCs [9,39,40]. Primary mMSCs obtained under 2% O_2 _were replated at 1,000/cm^2 ^and incubated for 3 to 4 days. At this stage, most of the cultured cells (designated as CmMSCs, see below) appeared as fibroblasts-like cells, similar to IDmMSCs cultured under 2% O_2_. Moreover their epitopes were also similar to those of IDmMSCs, negative for hematopoietic marker CD45 and CD11b, and endothelial marker CD31 and Flk-1, but positive for Sca-1 and CD44 (Figure 3B). Under appropriate conditions, CmMSCs at passage one were readily induced to differentiate into adipocytes, osteocytes and chondrocytes (Figure 3C). Together, culture of mMSCs under 2% O_2 _could successfully deplete most of contaminated hematopoietic cells, such that relatively pure mMSCs could be obtained at very early passage.

### MEF-ECM enhances proliferation of mMSCs

**Figure 4:**
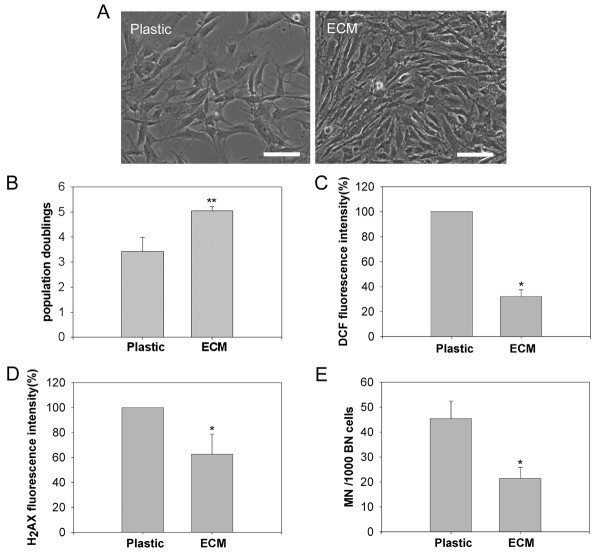
**Extracellular matrix derived from mouse embryonic fibroblasts (MEF-ECM) improves proliferation of mMSCs at early passage**. (A) Morphology of mMSCs at passage one cultured on plastic and MEF-ECM, respectively. (B) Population doublings of mMSCs cultured with or without MEF-ECM. mMSCs were plated at 1000 cells/cm^2 ^on 6-well dishes coated with or without MEF-ECM and incubated for 4 days, and cells were counted by hemocytometer. (C) ROS production by mMSCs cultured on plastic or MEF-ECM, measured by CM-H_2_DCFDA fluorescence. (D) Comparison of DNA double-strand breaks in mMSCs cultured on plastic or MEF-ECM, determined by flow cytometry analysis of γ-H_2_AX fluorescence intensity. (E) Spontaneous micronucleus formation in mMSCs cultured on plastic or MEF-ECM. Data are expressed as the mean ± SD. *p < 0.05; **p < 0.01; Scale bar = 100 μm.

Next, MEF-ECM was tested for proliferation capacity of mMSCs under 2% O_2_. As human marrow aspirates seeded directly on ECM lead to unwanted adherent of many other cells than MSCs [30], marrow cells were initially seeded on bare plastic, and then the primary mMSCs replated on MEF-ECM at 1000 cells/cm^2^. The growth rate of mMSCs on MEF-ECM (EmMSCs) was much higher than that cultured on plastic as control (CmMSCs). EmMSCs were more confluent, showing smaller cells, compared to CmMSCs at day 4 (Figure 4A), and the population doublings of EmMSCs were also greater than that of CmMSCs (Figure 4B). The identity of expanded cells cultured on MEF-ECM was confirmed by epitope analysis and differentiation assays (data not shown).

We further tested whether effects of MEF-ECM on expansion of mMSCs were associated with decreased oxidative damage. First, we measured levels of cellular reactive oxygen species (ROS) in mMSCs. ROS generated during normal oxidative metabolism may cause damage to DNA and contribute to genomic instability [41]. Cellular DCFDA fluorescence, indicative of relative ROS levels, was found significantly reduced in EmMSCs compared to CmMSCs (Figure 4C). We then evaluated the DNA double-strand breaks using flow cytometry analysis of γ-H_2_AX, which is routinely used to measure DNA damage caused by endogenous oxidants and other factors such as magnetic resonance imaging (MRI) exposure [42-44]. EmMSCs showed greatly decreased γ-H_2_AX intensity compared to CmMSCs (Figure 4D), suggesting reduced oxidative damage to mMSCs cultured on MEF-ECM. Reduced DNA damage was further revealed by micronucleus assay. While CmMSCs exhibited higher frequency of spontaneous micronucleus formation, EmMSCs showed greatly reduced MN (Figure 4E). Thus, mMSCs expanded on MEF-ECM showed reduced oxidative damage.

The above data show that combination of low oxygen and MEF-ECM can be used to obtain relatively pure proliferative mMSCs at early passages. We then examined whether the isolated mMSCs had normal karyotype. About 56% (28/50) metaphases prepared from EmMSCs at passage one had normal number of 40 chromosomes (Table 1), in contrast to reduced normal karyotypes from CmMSCs (30%, 15/50; Table 1), indicating a possible role of MEF-ECM in maintaining chromosome stability of mMSCs.

**Table 1:** Karyotypes of mMSCs cultured under 2% O_2 _

Number of chromosomes	< 40	40	41-79	80	> 80	Total number of metaphases	Normal karyotype (%)
**Passage 1**							
CmMSCs P1	3	15	13	2	17	50	30
EmMSCs P1	6	28	10	2	4	50	56

**Primary cultures**							
CmMSCs P0	2	12	15	8	13	50	24
PCmMSCs P0	5	31	6	2	6	50	62

PEmMSCs P0 (low density)	6	42	0	2	0	50	84

**Passage 8**							
CmMSCs P8	0	0	45	0	5	50	0
EmMSCs P8	1	0	43	2	4	50	0
PCmMSCs P8	1	1	2	0	46	50	2
PEmMSCs P8	3	14	29	1	3	50	28

Abbreviations: CmMSCs, mMSCs on control plastic; EmMSCs, mMSCs on MEF-ECM; PCmMSCs, mMSCs on control plastic in the presence of PBN and NAC; PEmMSCs, mMSCs on MEF-ECM in the presence of PBN and NAC.

### Antioxidants increase proliferation and chromosome stability of mMSCs

**Figure 5:**
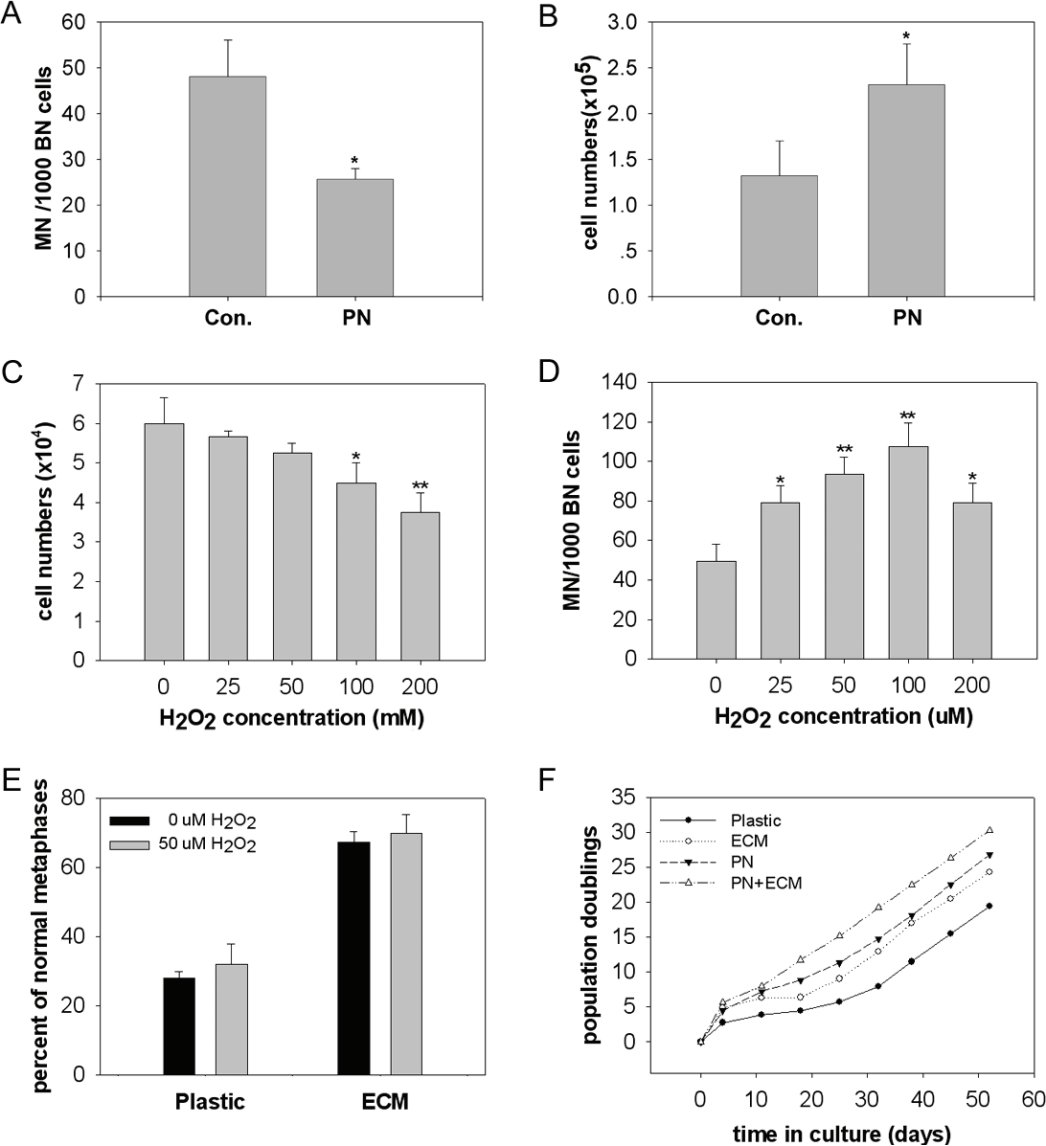
**Antioxidants increase proliferation of mMSCs**. (A) Comparison of spontaneous formation of micronucleus in primary mMSCs supplemented with or without antioxidants (800 μM PBN+5 mM NAC). (B) Effects of antioxidants on number of mMSCs. Bone marrow cells were plated at a density of 2 × 10^5 ^cells/cm^2 ^on 6-well and cultured under 2% O_2 _with or without addition of antioxidants for 5 days, and cells were counted by hemocytometer. (C) Effects of H_2_O_2 _on the proliferation of mMSCs. Primary mMSCs were replated at 1000 cells/cm^2 ^and cultured for two days, then H_2_O_2 _were added and cell number counted after 24 h. (D) Micronucleus analysis on mMSCs. (E) Karyotype analysis of mMSCs on plastic or ECM treated with H_2_O_2_. Data are expressed as the mean ± SD. *p < 0.05, **p < 0.01; n = 3. (F) A representative proliferation curve of mMSCs from three independent experiments. Data are expressed as the mean ± SD. *p < 0.05; n = 3. Abbreviations: Con., control; PN, PBN+NAC; CmMSCs, mMSCs on plastic as controls; EmMSCs, mMSCs on MEF-ECM; PCmMSCs, mMSCs on plastic in the presence of PBN and NAC; PEmMSCs, mMSCs on MEF-ECM in the presence of PBN and NAC.

The chromosome abnormalities in early passage mMSCs indicate that culture under low oxygen is not sufficient to protect mMSCs from oxidative damage. To further decrease possible accumulation of oxidative damage and therefore improve long-term viability of mMSCs, a combination of two antioxidants (800 μM PBN and 5 mM NAC) was used for initial isolation of mMSCs. PBN is a free radical spin trapping agent and has been shown to reduce oxidative damage and extend lifespan of human fibroblasts and peritoneal mesothelial cells [45,46]. NAC is an antioxidant that acts as the precursor of glutathione and as a scavenger of free radicals [47]. Antioxidants reduced number of spontaneous micronucleus (Figure 5A) and significantly improved proliferation (Figure 5B), and increased normal karyotype of primary mMSCs (Table 1), suggesting that antioxidants could effectively reduce oxidative damage to mMSCs.

To test whether artificial induction of oxidative stress increases chromosomal damages and abnormalities, we treated mMSCs for 24 hours with H_2_O_2 _at the concentrations that did not cause immediate cell death and found that H_2_O_2 _reduced cell proliferation (Figure 5C) and induced chromosomal damages as shown by increased rates of micronuclei (Figure 5D), despite that the concentration and treatment time of H_2_O_2 _used in this experiment did not cause appreciable abnormal karyotypes of mMSCs (Figure 5E).

We further evaluated effects of antioxidants on proliferation and ploidy of mMSCs following longer-term culture. mMSCs cultured without antioxidants showed a phase of slow growth at passage 3 and most of cells exhibited flat enlarged morphology like senescence, followed by gradual renewed growth possibly due to outgrowth of some actively growing colonies. In contrast, cells cultured with antioxidants continued proliferation. Notably, PEmMSCs (mMSCs on MEF-ECM in the presence of PBN and NAC) proliferated to passage 8 without obvious growth arrest (about 30 population doublings, Figure 5F). mMSCs at passage 8 proliferated actively and showed typical MSCs morphology as fibroblast-like cells. Yet, CmMSCs, EmMSCs or PCmMSCs (mMSCs cultured on plastic in the presence of PBN and NAC) at passage 8 showed nearly absent normal karyotype, whereas PEmMSCs at passage 8 still showed normal ploidy in 28% (14/50) of spreads analyzed (Table 1). These results indicate that combination of antioxidants and MEF-ECM might reduce oxidative damage to mMSCs to some extent and improve viability and chromosome stability of mMSCs for longer-term.

### Isolation of primary mMSCs with normal ploidy by seeding bone marrows cells directly onto MEF-ECM

**Figure 6:**
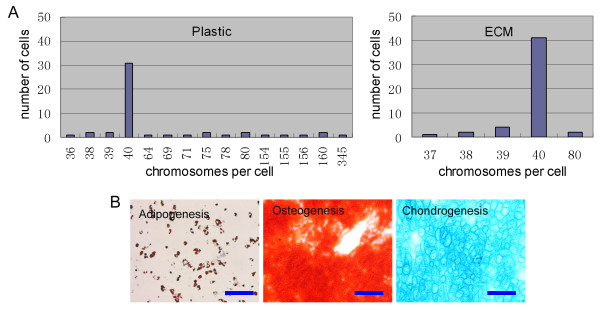
**Seeding of bone marrow cells directly onto MEF-ECM improves isolation of primary mMSCs with normal ploidy**. (A) Karyotype analysis of primary mMSCs cultured on plastic or MEF-ECM, at 2% O_2 _with addition of antioxidants. (B) The isolated mMSCs show differentiation potentials into typical cell lineages. Adipogenesis, osteogenesis, and chondrogenesis were indicated by Oil Red, Alizarin Red (pH 4.1) and Alcian blue, respectively.

Finally, we tested whether combination of antioxidants with MEF-ECM at the initial isolation step could increase mMSCs with normal karyotype. Bone marrow cells were seeded at a density of 2 × 10^5 ^cells/cm^2 ^in wells seeded with MEF-ECM and cultured as above. Cells cultured by day 5 showed mainly dense colonies composed of smaller spindle-shaped cells, but some dispersed small round cells outside dense colonies were also seen, possibly indicative of some contamination by hemaetopoietic cells. Notably, the isolated primary mMSCs showed normal karyotype in the majority (> 80%) of cells analyzed, while only 58% of mMSCs cultured on plastic as control had normal karyotype (Figure 6A). Further, bone marrow cells were seeded at a lower density of 5 × 10^4 ^cells/cm^2 ^to enrich more pure mMSCs. As a result, colonies composed of more pure spindle-shaped cells were obtained by day 6. Flow cytometry analysis revealed typical epitopes of mMSCs nearly free of hematopoietic cell contamination, and their tri-lineage differentiation potential into osteoblasts, chondrocytes, and adipocytes was also confirmed (Figure 6B). Importantly, most of these mMSCs exhibited normal ploidy (Table 1).

### Generation of induced pluripotent stem cells from mMSCs

**Figure 7:**
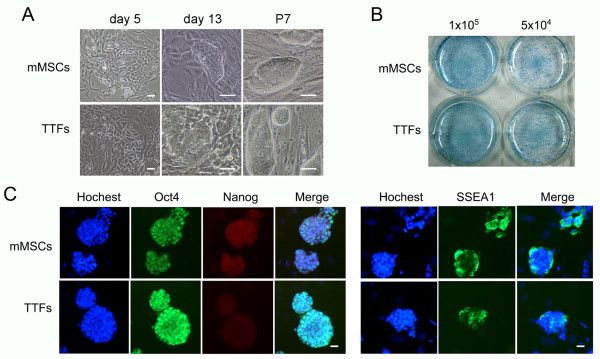
**Generation of induced pluripotent stem cells from mMSCs**. (A) Morphology of iPS cells from mMSCs and TTFs (B) Alkaline phosphatase positive clone formation from mMSCs and TTFs. mMSCs and TTFs were plated at 1 × 10^5 ^cells/well or 5 × 10^4 ^cells/well and were infected with virus/polybrene-containing supernatants, alkaline phosphatase assay was performed 11 days after transduction. (C) Immunofluorescence analysis of pluripotency-associated transcription factors and surface marker in mMSCs-iPS and TTFs-iPS at passage 7. Scale bar = 100 μm.

We next tested whether mMSCs isolated by the new combination method could be used to generate iPS cells. By transfection of mMSCs with four Yamanaka's factors carried by retroviral vectors, we successfully derived iPS cells from mMSCs. mMSCs generated alkaline phosphatase-positive colonies with dynamics and efficiency similar to those of tail-tip fibroblasts (TTFs) (Figure 7A, B). Forty eight such colonies were picked and passaged for both mMSCs and TTFs cultures, three such subcultures of mMSCs and two of TTFs expanded and gave rise to stable cell lines (passaged for at least seven passages) with morphology and characteristics of mouse ES cells. It seemed that mMSCs generated iPS cell-like colonies by day 13, little earlier than did TTFs. To validate mMSCs derived iPS cells, the expression of pluripotency-associated transcription factors and surface marker was analyzed by immunofluorescence microscopy, and mMSCs-iPS cells expressed Oct4, Nanog and SSEA1, similar to TTFs-iPS cells (Figure 7C).

## Discussion

MSCs are routinely cultured under 20% O_2 _in DMEM or α-MEM, supplemented with 10-20% fetal bovine serum. Isolation and long-term culture of mouse MSCs under these classical culture conditions has been found problematic, compared to that of other species [9]. Consistent with previous studies [19], our data show that normal ploidy mMSCs are rarely achieved despite of enormous efforts to optimize culture conditions under 20% O_2_. However, mMSCs can be purified simply by culture under 2% O_2_, mainly attributable to significantly improved proliferation of mMSCs and reduced oxidative damage. Also, passaging by harvesting only easily detached cells without pipetting [9,48] and seeding at a relatively low density of 1000 cells/cm^2 ^improves purification and proliferation of mMSCs, such that a purer population of mMSCs could be obtained only 9 days after initiation of culture.

ECM prepared from MEF further expands mMSCs and reduces oxidative damage. This might be explained by more undifferentiated state and higher antioxidant defence capacity of mMSCs expanded by MEF-ECM. Stress defence of stem cells is usually superior to that of differentiated cells [49], and ECM could maintain higher differentiation potential of multipotent MSCs than that of bare plastic [30,31,33,50]. We did not quantitatively compare the differentiation potential of mMSCs expanded on plastic and MEF-ECM, but the smaller size may imply a more undifferentiated state of mMSCs expanded by MEF-ECM. Thus, combination of low oxygen and MEF-ECM might provide an effective strategy for obtaining relatively pure mMSCs at more undifferentiated state and with less oxidative damage at early passage.

However, mMSCs cultured on either plastic or MEF-ECM show high frequency of chromosome abnormalities. Further, chromosome abnormalities are found just upon primary *in vitro *culture of bone marrow cells. Consistently, several groups have already reported chromosome abnormalities in bone marrow derived mMSCs cultured under 20% O_2 _[13,15-19]. Recently, adipose tissue-derived mMSCs under long-term culture also exhibit chromosomal abnormalities [51], suggesting mouse species-specific sensitivity to oxidative damage and chromosomal instability [22]. Chromosome abnormalities found in early passage mMSCs under 2% O_2 _suggest that low oxygen alone or in combination with MEF-ECM is insufficient to protect mMSCs from oxidative damage and chromosome instability. But increased rates of normal chromosomes found in EmMSCs than in CmMSCs suggest a possible role of MEF-ECM in reducing chromosome instability of mMSCs.

Antioxidants might confer mMSCs additional antioxidant defence capacity. A combination of two antioxidants (PBN and NAC) significantly reduces spontaneous MN and increases normal karyotype of primary mMSCs, in association with increased proliferation of mMSCs. Particularly, mMSCs could be expanded continuously on MEF-ECM without noticeable growth arrest for at least 8 passages (about 30 population doublings, Figure 5F). One-third of PEmMSCs still exhibit normal ploidy by passage 8, in contrast to nearly absent normal ploidy found in CmMSCs, EmMSCs and PCmMSCs, suggesting that antioxidants in combination with MEF-ECM improve proliferation and chromosomal stability of mMSCs.

Together, a method to isolate mMSCs with normal ploidy is developed by combination of low oxygen, antioxidants and MEF-ECM. Consistent with another study [30], directly seeding marrow cells on MEF-ECM leads to contamination of a small fraction of hemaetopoietic cells. This problem can be partly resolved by lowering seeding density from 2 × 10^5 ^cells/cm^2 ^to 5 × 10^4 ^cells/cm^2^. Seeding at low density has been adopted to obtain pure primary mMSCs [39,40]. As a result, a purified population of mMSCs with normal ploidy can be obtained only 6 days after initiation of primary culture.

The quality of mMSCs isolated by the new combination method was further tested by their potential to generate iPS cells. iPS cells have been induced from various types of mouse cells, including fibroblasts [52], adipose tissue-derived cells and neural stem cells [53], and hepatocytes and gastric epithelial cells [54]. Poor quality of mMSCs may hinder their reprogramming process of iPS cells. With limited experiments, we successfully generated iPS cells from mMSCs isolated by our new method with at least comparable efficiency to TTFs, one of the most commonly used donor cells for iPS induction [52]. We found that iPS cell like colonies formed earlier from mMSCs than from TTFs, suggesting that mMSCs could be prone to reprogramming. However, more studies are needed to determine whether the resulted iPS cells from mMSCs show more complete reprogramming and higher quality than from other cell types, but successful generation of iPS cells from mMSCs validated the effectiveness of our new method. On the other hand, accessibility in clinics of somatic cells for iPS induction is a consideration. Our method can be adaptable to human MSCs derived from bone marrow aspirations, since both low oxygen and ECM are shown to benefit for growth of human MSCs [24,30]. hMSCs cultured by the new method could be appropriate somatic cells for induction of iPS cells for potential therapeutic uses.

## Conclusions

We have developed a method for isolation of relatively pure primary mMSCs with normal ploidy by combination of low oxygen, MEF-ECM, antioxidants and low density plating strategy. mMSCs isolated by this method were successfully used to generate iPS cells. These normal ploidy mMSCs could also be useful for numerous other studies in the mesenchymal stem cell field. Our data also suggest that conditions for long-term culture of mMSCs should be developed to maintain normal ploidy mMSCs for future uses.

## Methods

### Preparation of MEF-ECM-coated wells

MEF was isolated from 13.5 dpc embryos of C57BL/6 × DBA/2 hybrid background and maintained as described [55]. For preparation of MEF-ECM-coated wells, confluent passage one MEF cultures in 6 well plates were allowed to continue for another day. The cultured cells were treated with 20 mM NH_3 _and 0.5% Triton X-100 for 5 min at room temperature, the lysis buffer was removed, and the plates were placed without cover for 20 min to allow the ECM adhere firmly to the substrata of the wells. The substratum was washed five times with Hanks' balanced salt solution (HBSS; Invitrogen) and stored at 4°C for subsequent uses.

### Isolation and expansion of mMSCs

Young B6D2F1 mice (3-5 weeks old) were sacrificed by cervical dislocation using protocol approved by the Institutional Animal Committee. Whole bone marrow was harvested by flushing femurs and tibiae as described [9]. The marrow cells were plated into 6-well cell culture plate at a density of 2 × 10^5 ^cells/cm^2 ^in 2.5 ml DMEM containing 20% fetal bovine serum (Hyclone) and 1% penicillin-streptomycin antibiotics (Invitrogen). In some experiments, a combination of antioxidants (800 μM alpha -phenyl-t-butyl nitrone (PBN) and 5 mM N-acetylcysteine (NAC)) were added in the medium. In others, bone marrow cells were seeded directly onto MEF-ECM-coated wells. Cells were cultured at an atmosphere of 2% O_2 _(2% O_2_, 93% N_2_, 5% CO_2_) at 37°C. Non-adherent cells were removed after 24 hours by washing twice with phosphate-buffered saline (PBS), and fresh medium added. At day 5-6, adherent cells were treated with 0.25% trypsin/0.02% EDTA (Invitrogen) for 3 min at 37°C. The cells lifted within 3 min were collected and replated at 1000 cells/cm^2 ^onto uncoated 6-well plates or plates coated with MEF-ECM. The medium was replaced every 2-3 days until cells reached 90% confluence. Subsequent passaging was carried out using the same conditions.

For initial experiments to optimize culture conditions under 20% O_2_, the marrow cells were plated at a higher density of 2 × 10^6 ^cells/cm^2^, and subcultures were performed at a split ratio of 1:3 when cells reached 90% confluence.

For immunodepletion experiments, retrieved primary mMSCs were stained with PE-conjugated anti-CD45 and anti-Ter119 antibodies (Miltenyi Biotec), and then stained with anti-PE magnetic microbeads (Miltenyi Biotec) and immunodepeleted according to the manufacturer's instruction. Cells negative for CD45 and Ter119 were seeded onto 6-well plates at 10, 000 cells/cm^2^.

### *In vitro *Differentiation of mMSCs

To induce osteogenic differentiation, 70-80% confluent mMSCs were incubated in osteogenic medium (DMEM supplemented with 20% FBS, 20 mM β-glycerol phosphate, 1 nM dexamethasone and 0.5 μM ascorbate 2-phosphate) for one week with medium changed once, based on a method with slight modification [9]. The cells were fixed with 3.7% paraformaldehyde and stained with Alizarin Red (pH 4.1).

To induce adipogenic differentiation, 70-80% confluent mMSCs were incubated in adipogenic medium (DMEM supplemented with 20% FBS, 0.5 μM hydrocortisone, 0.5 mM isobutylmethylxanthine (IBMX) and 60 μM indomethacin [56], for one week and the medium was changed once. The cells were fixed in 3.7% paraformaldehyde and stained with 0.5% Oil Red in propylene glycol.

Chondrogenic differentiation was performed in 70-80% confluent monolayer or in a pellet [9] formed by centrifugation of 2 × 10^5 ^mMSCs. The chondrogenic medium consisted of high-glucose DMEM supplemented with 500 ng/ml bone morphogenetic protein-2 (BMP-2; Peprotech), 10 ng/ml transforming growth factor β3 (TGF-β3; Peprotech), 10^-7 ^M dexamethasone, 50 μg/ml ascorbate-2-phosphate, 40 μg/ml proline, 100 μg/ml pyruvate, 1 × insulin-transferrin-selenium and 1 × lenolenic acid-bovine serum albumin (LA-BSA) (Sigma). The monolayers or pellets were incubated in this medium for 1-2 weeks with medium change twice a week. The monolayers were fixed and stained with Alcian blue. The pellets were embedded in tissue-freezing medium (Leica), sectioned into 8-μm slices, fixed with cold acetone and stained with Alcian blue.

### Immunostaining and FACS analysis

For flow cytometry analysis of mMSCs epitope, following PE conjugated antibodies were used: CD31, CD34, Flk-1, Sca-1, CD44 (eBioscience), CD45, CD11b (Miltenyi Biotec). Cells were divided into aliquots (5 × 10^4 ^each), stained with PE-conjugated antibodies at final concentration of 2 μg/ml at room temperature for 30 min, washed with PBS and analyzed by flow cytometry. For flow cytometry analysis of γ-H_2_AX, cells were fixed with 3.7% paraformaldehyde, permeabilized with 0.1% Triton X-100 and blocked in 3% normal goat serum (Sigma) in PBS. After incubation with 2 μg/ml anti-γ-H_2_AX antibody (Upstate, 05-636) at 4 °C overnight, the cells were washed and stained with Alexa 488-conjugated goat anti-mouse IgG (1:100 v/v, Molecular Probes) for 1 hour, and washed and analyzed as above. Immunostaining of pluipotency-associated markers was performed with following primary antibodies: Oct-4 (sc5279, Santa Cruz, CA), Nanog (Abcam, ab10626) and SSEA1 (DSHB, MC-480), and secondary antibodies: Texas red conjugated anti-mouse IgG (Vector, TI-2000, CA) or Alexa fluor 488 goat anti-mouse IgM (Molecular Probes). Immunofluorescence was observed and imaged using a Leica microscope equipped with epi-fluorescence and appropriate filters.

### ROS detection

Intracellular reactive oxygen species (ROS) was determined by flow cytometry using 5-b-chloromethyl-2'7'-dichlorodihydrofluorescin diacetate (CM-H_2_DCFDA; Molecular Probes) [49,57]. mMSCs were harvested by trypsin-EDTA and incubated in serum-free medium containing 10 μM CM-H_2_DCFDA for 30 min at 37°C. Then the cells were washed, resuspended, and analyzed by flow cytometry. The median fluorescence intensity was used to estimate intracellular ROS levels.

### Micronucleus (MN) assay

Micronucleus assay was performed as described with slight modification [58]. Cultured mMSCs at 50-80% confluence were incubated with 4.5 μg/ml cytochalasin-B (Sigma) for 24 hours and harvested. After a brief hypotonic treatment in 0.075 M KCl, cells were fixed in methanol/acetic acid (5:1), stained with 5 μg/ml Hoechst 33342 (Molecular Probes) in PBS and mounted for immunofluorescence microscopy. Frequency of micronucleus was assessed on 1000 binucleated cells for each culture in three independent experiments.

### Karyotype analysis and telomere fluorescence in situ hybridization (FISH)

mMSCs at 50-80% confluence were incubated with 0.1 μg/ml colcemid (Sigma) for 5 h. The cells were incubated in hypotonic solution (0.075 M KCl), fixed in methanol/acetic acid (3:1) and spread onto slides. Cell spreads were stained with 5 μg/ml Hoechst 33342 (Molecular Probes) in PBS, examined by immunofluorescence microscope, and number of chromosomes were counted. FISH with FITC-labelled telomere-specific (CCCTAA)_3 _peptide nucleic acid (PNA) probe (Applied Biosystems, Framingham, MA, USA) was performed as described [56].

### Tumorigenesis assay

2 × 10^6 ^mMSCs were subcutaneously injected into 4 weeks old BALB/c nude mice. The animals were monitored for tumor formation for two months. Formed tumors were excised, fixed in formalin, paraffin embedded, sectioned, and stained with hematoxylin and eosin.

### Generation of iPS cells

iPS cells were induced by transduction of four Yamanaka's factors according to the protocol of Okita et al. [59] with slight modifications. Briefly, pMXs-based retroviral vectors (pMXs-Sox2, Klf4, Oct4, c-Myc) were introduced into Plat-E cells using Lipofectamine 2000 (Invitrogen) according to the manufacturer's recommendations. The viral supernatant were collected, filtered (0.45 μm pore size) and supplemented with 8 μg/ml polybrene before use. The day before infection, primary mMSCs and tail-tip fibroblasts (TTFs) were plated at 5 × 10^4 ^cells/well into 6-well plates coated with MEF-ECM or gelatin. The mMSCs and TTFs were infected with virus/polybrene-containing supernatant twice within forty eight hours. Three days after infection, the cells were replated in ES medium (knock-out DMEM supplemented with 20% FBS, 1000 U/ml LIF, 0.1 mM β-mercaptoethanol, 1 mM L-glutamine and 0.1 mM nonessential amino acids). The cells were passaged at day 5 onto MEF feeders and the medium was changed every day. ES-like colonies were picked by day 10 to 13, and passaged onto MEF feeders.

### Alkaline phosphatase staining

Alkaline phosphatase assay was performed using the Vector Blue Aalkaline Phosphatase Substrate Kit (Vector Laboratories, DAKO, Carpinteria, CA) according to manufacturer's instruction.

### Statistical analysis

The data were analyzed by Student's t-test. Results are expressed as mean ± SD. A value of p < 0.05 was considered significant by comparison. All experiments were performed at least three times.

## List of abbreviations used

mMSCs: mouse mesenchymal stem cells; ECM: extracellular matrix; IDmMSCs: immunodepleted mMSCs; PBN: alpha -phenyl-t-butyl nitrone; NAC: N-acetylcysteine; CmMSCs: mMSCs on control plastic; EmMSCs: mMSCs on ECM; PCmMSCs: mMSCs on control plastic in the presence of PBN and NAC; PEmMSCs: mMSCs on ECM in the presence of PBN and NAC. TTFs: tail-tip fibroblasts; iPS cells: induced pluripotent stem cells.

## Authors' contributions

FGK conducted the majority of experiments and contributed to drafting the manuscript. WL participated in cell culture experiments and micronucleus assay. LC participated in tumorigenic assay and performed the statistical analysis. LBP participated in karyotype analysis. WF aided in MEF preparation and participated in differentiation experiments. LMS and WF conducted iPS and related experiments. ZLJ provided support and participated in FACS analysis. LL conceived the study and contributed to drafting the manuscript. All authors read and approved the final manuscript.
